# Wild Pepper (*Piper laetispicum*) Fruit Quality Traits at Different Developmental Stages

**DOI:** 10.3390/molecules29174008

**Published:** 2024-08-24

**Authors:** Zhican Zhao, Yiming Fang, Dan Zhang, Jue Wang, Yanli Huang, Chaoyun Hao, Rui Fan

**Affiliations:** 1 Tropical Croups Genetic Resources, Chinese Academy of Tropical Agricultural Science (CATAS), Haikou 571101, China; 2020314993@stu.ynau.edu.cn (Z.Z.); fangym1987@catas.cn (Y.F.); 2 College of Tropical Crops, Yunnan Agricultural University, Pu’er 665099, China; 2020315159@stu.ynau.edu.cn (D.Z.); 2008074@ynau.edu.cn (Y.H.); 3 Spice and Beverage Research Institute, Chinese Academy of Tropical Agricultural Science (CATAS), Wanning 571533, China; 18434161750@163.com; 4 Key Laboratory of Genetic Resources Utilization of Spice and Beverage Crops, Ministry of Agriculture, Wanning 571533, China

**Keywords:** *Piper laetispicum*, volatile oil compounds (VOCs), aroma metabolite, GC-MS

## Abstract

High-quality *Piper laetispicum* (*Piper laetispicum* C. DC) is the key to the development of foods, natural medicines, and cosmetics. Its crude fat, ash, piperine, protein, and aroma compounds were determined in this experiment. Principal component (PCA) and hierarchical cluster analyses (HCA) were used to evaluate the aroma compounds at different developmental stages. The main aroma compounds identified using steam distillation combined with GC-MS were sabinene (34.83–76.14%), *α*-copaene (5.11–19.51%), linalool (2.42–15.70%), trans-caryophyllene (2.37–6.57%), *α*-pinene (1.51–4.31%), and germacrene D (1.30–4.10%). The aroma metabolites at different developmental stages were analysed using non-targeted metabolomes, and linalool was found to be the most abundant. Based on the experimental results, there were more nutrient compounds in young *Piper laetispicum* than in the last three developmental stages. The aromatic metabolites contributed the most to PC1. There were also more different metabolites of aroma between the young and expanding stages. Therefore, regarding quality, young fruits have great potential.

## 1. Introduction

*Piper laetispicum* C. DC. (Piperaceae), a wood-climbing vine and a wild species of piper, is commonly known as mountain or wild pepper and widely used in traditional Chinese medicine to treat small intestine bloating. It grows in the jungles in Guangdong, Guangxi, and Hainan Provinces, China. It can improve blood circulation, remove blood stasis, reduce swelling, and relieve pain. In Dai medicine, boiling and consuming the entire plant positively impacts heart problems, stomach and abdominal pain, diarrhoea, and indigestion [1,2,3]. Laetispicine from *P. laetispicum* stems is a new amide alkaloid that can promote the production and development of new antidepressants, which are used to help people overcome depression [4].

*Piper nigrum* L. is the “king of spices” and has attracted widespread interest from researchers. It has a flavour and serves as a medicinal resource for relieving nerve pain and the treatment of influenza and fever, with both anti-bacterial and anti-inflammatory activities [5,6]. There are reference standards for the quality trait evaluation of pepper, its genome, and the utilisation and preservation of the effective parts of pepper. The flavour and unique aroma of pepper are mostly dependent on piperine and the aroma ingredients [7]. The volatile compounds of 10 pepper varieties cultivated in Hainan Province, China, mainly include benzaldehyde, cinnamaldehyde, *β*-caryophyllene, ocimene, lavandulol, myrcene, cubebene, terpinene, linalool, *α*-caryophyllene, *β*-elemene, and germacrene. These compounds determine the bitterness and astringency of pepper [8]. The active compounds extracted from *P. laetispicum* have attracted much attention. For example, the antidepressant efficacy of Laetispicine was determined [4,9]. However, few studies have characterised the chemical composition of *P. laetispicum* fruit. 

Essential oils have a pleasant taste and smell and have antioxidant, anti-corrosion, and other effects. The chemical and functional properties and composition of volatile oil compounds extracted using steam distillation are important [10,11]. The function of the compounds of the volatile oil of *P. laetispicum* was elucidated using steam distillation and GC-MS analysis. Metabolites can be divided into primary and secondary ones, which affect the growth and development of plants and their responses to the surrounding environment [12]. The aim of metabolomics is to determine the biological processes cells and tissues undergo by analysing metabolite changes over time. Non-targeted metabolomes systematically and comprehensively represent material information for the detection and analysis of endogenous metabolites [13]. The aroma compounds of *P. laetispicum* were analysed at four developmental stages based on non-targeted metabolomes. This is conducive to understanding the diversity, functions, and pathways of natural metabolites, ensuring food health and safety. It also promotes the development and application of plant metabolic engineering in the production of drugs and new materials [14,15,16]. 

The aroma components in the fruit of *P. laetispicum* have been determined by many scientists and technologists. The aim of this study was to determine the composition and content of the resulting compounds and to screen and analyse their differential metabolites using non-targeted metabolomic methods. The biological significance of the metabolites at different developmental stages was clarified through the analysis of metabolic pathways. We not only comprehensively analysed the aroma components of fruits but also further explored the differential metabolic pathways of the aroma components. Finally, we provide data for the aroma characteristics of *P. laetispicum* to inform the selection, processing, and growth of fruits in different harvesting periods.

## 2. Results and Discussion

### 2.1. Crude Fat, Ash, Piperine and Protein Contents

As shown in Table 1, the contents of crude fat, ash, and protein in the young and expanding fruit stages were minimally different, and the contents were at the highest in the whole fruit stage. The contents in the yellow fruit stage showed a decreasing trend. In addition to the continuous decline in the crude fat content in the red fruit stage, the protein and ash contents slightly increased. The content of piperine was very low in *P. laetispicum* fruits (Figure A5).

### 2.2. VOCs 

#### 2.2.1. Steam Distillation Combined with GC-MS Analysis of VOCs 

**Figure A1:**
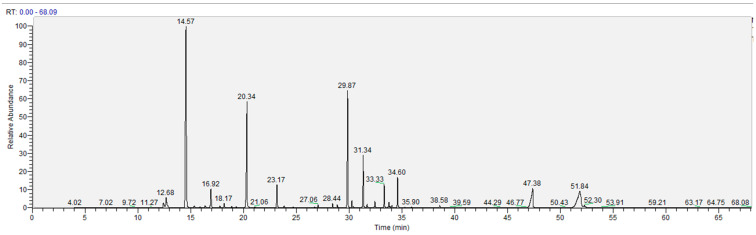
Ion map of VOCs of *P. laetispicum* at young fruit stage.

**Figure A2:**
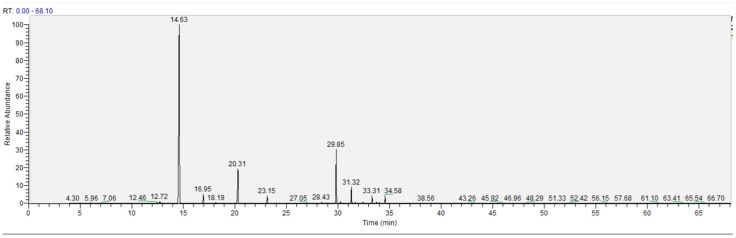
Ion map of VOCs of *P. laetispicum* at expanding fruit stage.

**Figure A3:**
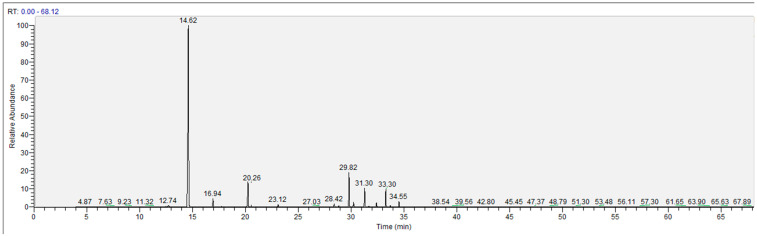
Ion map of *P. laetispicum* VOCs at yellow fruit stage.

**Figure A4:**
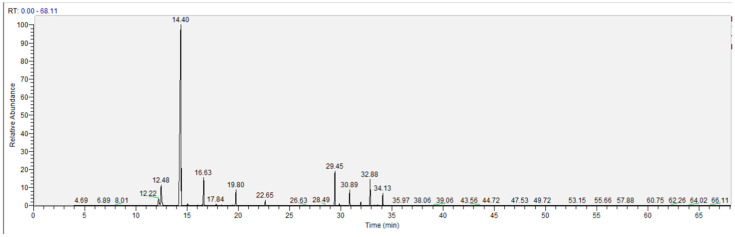
Ion map of VOCs of *P. laetispicum* at Red fruit stage.

**Figure A5:**
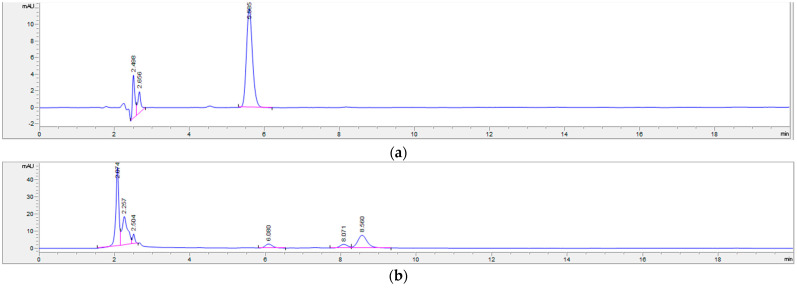
An HPLC diagram of piperine content. (**a**) Standard piperine content. (**b**) The piperine content of *P. laetispicum* at the yellow fruit stage.

A total of 49 compounds were identified in volatile oil at the four stages, including young (34), expanding (30), yellow (30), and red (37) fruits. The functional group had five categories: alkenes, alcohols, ketones, esters, and benzene (Table 2 and Table A2, and Figure A1, Figure A2, Figure A3 and Figure A4). Alkenes and alcohols contributed more to the *P. laetispicum* aroma, with lower contents of ketones, benzene, and esters. The contents of the following aromatic compounds were high: sabinene (34.83–76.14%), *α*-copaene (5.11–19.51%), linalool (2.42–15.70%), trans-caryophyllene (2.37–6.57%), *α*-pinene (1.51–4.31%), and germacrene D (1.30–4.10%). 

Sabinene is a monoterpene with an anti-inflammatory effect. This substance was detected in *Piper nigrum*, but there was only approximately 2% [17]. Its contents in “Jinguangshanshuo”, “Bingsham”, and “Honglou” were 5.87%, 4.61%, and 11.07%, respectively [18]. The relative contents of the *P. laetispicum* fruits in the four development stages were all between 30% and 80%, which was higher than that in most pepper plants. This may be one of the main factors for its utilisation as a Chinese medicine resource. Linalool has a fresh floral scent and self-oxidises in the air to facilitate the production of hydroperoxide, which is a strong allergen [19,20]. Trans-caryophyllene is the most representative compound in the essential oils of pepper fruits and has a spicy, woody, citrus, and camphor taste [21]. *α*-pinene is abundant in turpentine. Perfumers have a special interest in it, and it also has significant anti-tumour and anti-cardiotoxicity activities [22]. 

The following list is of specific compounds at the young fruit stage and their uses: (*Z*)-linalool oxide (furanoid) has a strong woody flower aroma and is a commonly used chemical flavouring. *α*-bisabolol sesquiterpene alcohol, with anticancer activity, has an anti-ageing effect and can treat inflammatory acne [23]. Guaiazulene is a special anti-inflammatory ingredient in essential oils and is only present at the red fruit stage. Its strong oxidation has great potential in the field of medicine [24,25]. *P. laetispicum* fruit is rich in aroma compounds with analgesic, anti-inflammatory, and anticancer effects. In conclusion, it also has excellent potential in the development of natural drugs, in addition to the production of cosmetics and perfumes. 

The content of alcohols gradually decreased as the *P. laetispicum* fruit matured, while the content of alkenes gradually increased. Alcohol accumulation in Cabya mainly occurs at the expanding and yellow fruit stages [26], while it slows down at the young and red fruit stages. The relative content of alcohols is different between different species of pepper and in fruits at different developmental stages. This may be due to the differences in the growing environment or the growth pattern of the plant [27]. The alkenes in Cabya were found to be enriched at the red fruit stage, indicating that alkenes may contribute to most to the aroma of the most ripe pepper fruits. 

#### 2.2.2. Principal Component Analysis (PCA) of VOCs

The VOCs were analysed using PCA (Table 3 and Table 4). The first three principal components with eigenvalues greater than one were selected, and the cumulative contribution rate reached 100%, indicating that the three principal components comprehensively characterised all the information of the 49 compounds. The contribution rate of principal component 1 was 54.67%. Alcohols, alkenes, ketones, and esters, the characteristic volatile components of the fruit, all had large positive and negative loads. The contribution rate of principal component 2 was 28.36%. The *β*-myrcene load coefficient was −0.98. *β*-myrcene has antioxidant, anti-bacterial, and analgesic effects and is found in many plants, including lemongrass, hops, verbena, and bay. It is often used as a raw material in the production of cosmetics [28]. Principal component 2 reflected the aroma volatiles of some monoterpenes. The contribution rate of principal component 3 was 16.98%. The maximum load coefficient of *α*-caryophyllene was 0.917, and it is a colourless or micro-buttery liquid with a faint butyl flavour. Principal component 3 reflected some sesquiterpenes.

**Figure 1:**
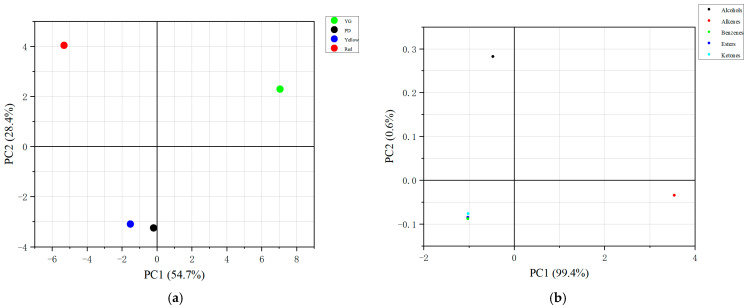
Principal component analysis (PCA) of 49 VOCs detected in *Piper laetispicum* fruit at four developmental stages. (**a**) Scatter plot of PCA scores for each phase; (**b**) scatter plot of PCA scores for VOC types.

The young fruit stage is shown in the upper right corner of the score chart (Figure 1a), while the red fruit stage is represented in the upper left corner. The difference in aromatic compounds between the young and red fruit stages was the most significant. Alkenes were found to have a highly positive correlation with principal component 1, and alcohols had a highly positive correlation with principal component 2 (Figure 1b).

#### 2.2.3. Cluster Analysis of VOCs

**Figure 2:**
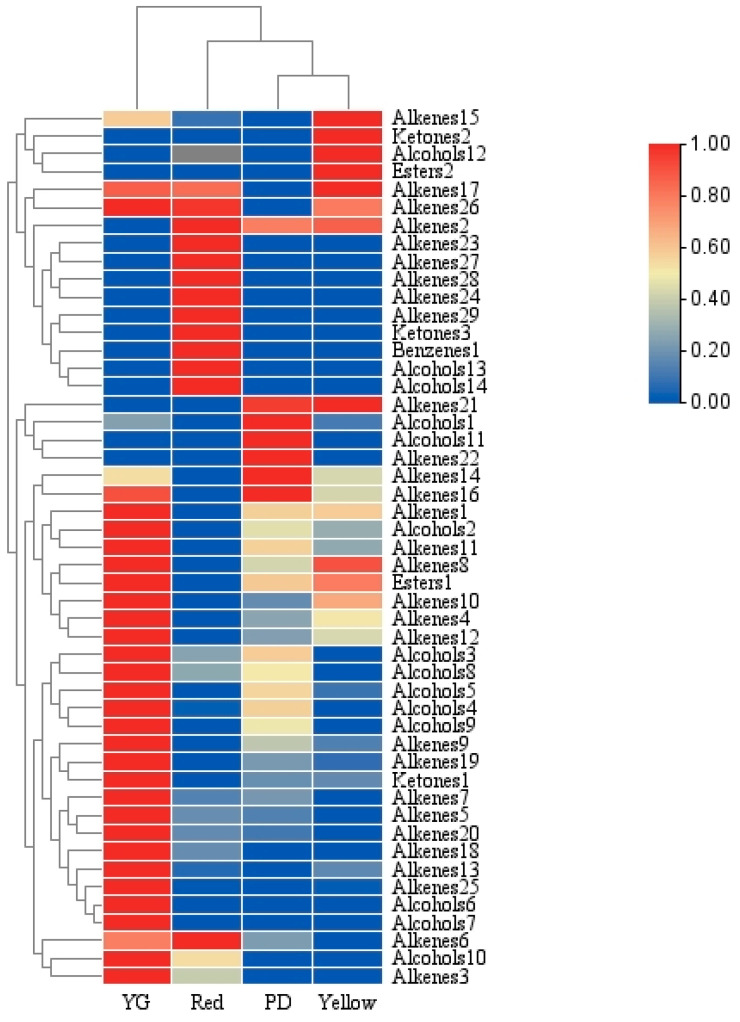
Hierarchical cluster analysis (HCA) of the four *Piper laetispicum* fruit colour stages based on the contents of 49 VOCs.

The four samples were classified into three categories based on the aroma components, among which the expanding and yellow fruit stages were similar and were placed in the same category. In subsequent clustering, the expanding, yellow, and red fruit stages were grouped together. The samples in the young fruit stage were found to be significantly different from those from the other groups (Figure 2), which is consistent with the results of the score charts (Figure 1a).

### 2.3. Aroma Metabolites

#### 2.3.1. Identification of Aroma Metabolites

**Figure 3:**
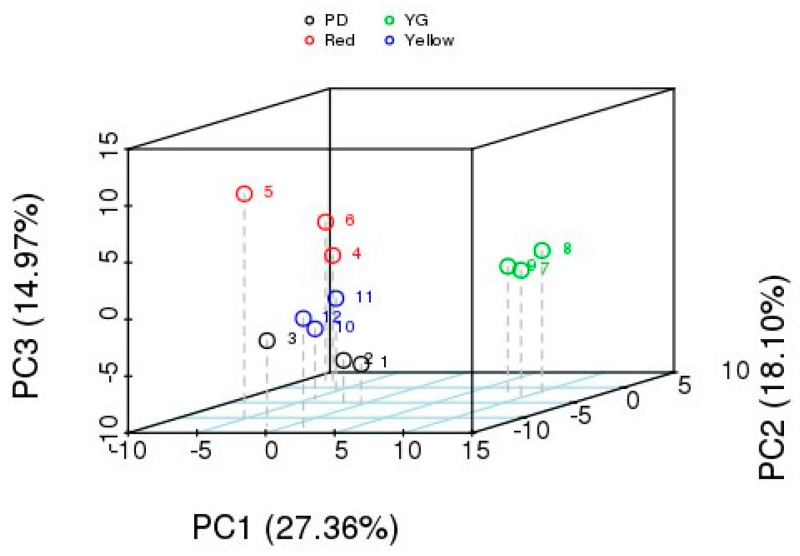
PCA analysis of total samples.

The fruit aroma metabolites in the biological repeats were clustered together, indicating that the duplicate sample was stable and reliable. The fruits at the different stages of development were distinct (Figure 3), suggesting that there were some differences in metabolites between them. A total of 143 metabolites from the *P. laetispicum* fruit were identified with non-targeted metabolome analysis using GC-MS. Linalool, copaene, sabinene, germacrene D, *β*-cadinene, (−)-*β*-pinene, and *α*-pinene, (+)- were the main metabolites in *P. laetispicum* fruits. The linalool content was the highest among them (Table A2). PCA found that the main components accounted for 60.43%. The young fruit was significantly different from the expanding, yellow, and red fruits, which was consistent with the GC-MS results of the volatile oil.

#### 2.3.2. Identification and Analysis of Differential Aroma Metabolites

**Figure 4:**
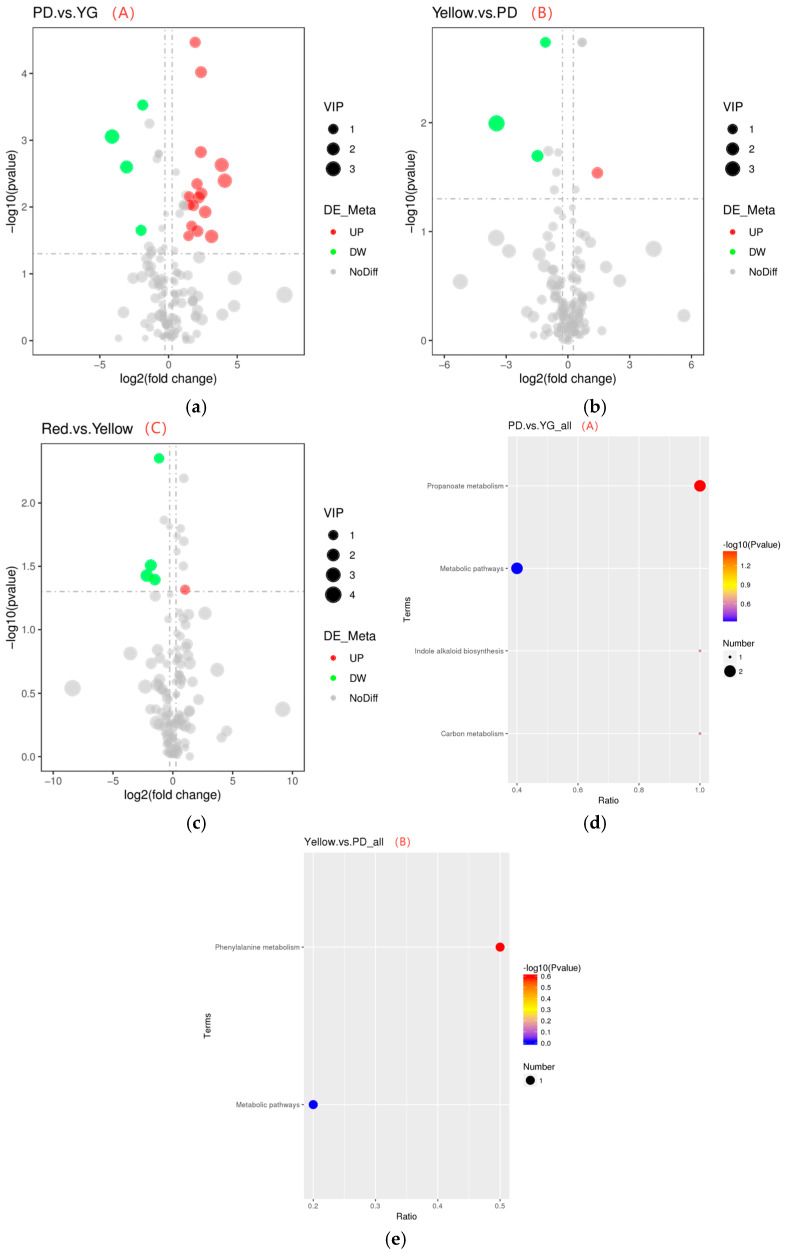
A, B and C represent fruit growth and development from the young to expanding stages, from the expanding to yellow fruit stages, and from the yellow fruit to red fruit stages, respectively. (**a**–**c**) A differential metabolic volcano map. The horizontal coordinates represent the change in the levels of metabolites in the different groups (log_2_FoldChange), and the vertical coordinates represent the significance level of the differences (−log_10_p-value). Each point in the volcanic map represents a metabolite. The red dots represent the significantly upregulated metabolites, and the green dots represent the significantly downregulated metabolites. The dot size represents the VIP value. (**d**,**e**) A KEGG-enriched bubble diagram. The horizontal coordinate is x/y (the number of differential metabolites in the corresponding metabolic pathway/the total number of identified metabolites in the pathway). A higher value indicates a higher concentration of differential metabolites in the pathway. The colour of the dots represents the *p*-value of the hypergeometric test, and a smaller value represents a more reliable and statistically significant test. The dot size represents the number of differential metabolites in the corresponding pathway, and a larger point represents more differential metabolites in the pathway.

Metabolome analysis based on GC is useful for describing the changes in metabolites at different stages [29]. In this experiment, the fold change (FC) in pepper fruit in the comparison group, the p-value obtained using the t-test, and the variable projection VIP value obtained using PLS-DS were used to screen the different metabolites. The differential metabolites must have FC > 1.2, VIP > 1.0, and *p* < 0.05. A total of 41 differential metabolites were identified in *P. laetispicum* fruit at the four stages. “A” had the most differential metabolites, with four downregulated metabolites and sixteen upregulated metabolites; “C” took second place, with four downregulated metabolites and one upregulated metabolite; and “B” had the least, with three downregulated metabolites and one upregulated metabolite. As the fruits developed and ripened, the metabolites gradually changed, which was the most obvious from the young to the expanding fruit stages, with most metabolites being upregulated. After the expanding fruit stage, the metabolites changed more slowly (Figure 4a–c). The quantitative analysis results show that the content of the differential metabolite trans-2-pentenal gradually decreased from the young to the red fruit stages (Table A2). Trans-2-pentenal is mainly found in spices and has a significant inhibitory effect on cancer cell growth and almost no effect on normal cells [30].

#### 2.3.3. Metabolic Pathways of Differential Aroma Metabolites

According to the KEGG database, at *p* < 0.5, the enrichment pathways of the differential metabolites of “A”, “B”, and “C” were analysed. As shown in Figure 4d,e, the differential metabolites of “A” were involved in propionate and carbon metabolism and indole alkaloid synthesis, while the differential metabolites of “B” were involved in phenylalanine metabolism. In these metabolic pathways, the differential metabolites methanol and propionaldehyde were upregulated, and phenacetaldehyde was downregulated. Propionate [31], phenylalanine [32], carbon metabolism [33], and indole alkaloid synthesis [34] are important metabolic pathways for the development of *P. laetispicum* fruit.

## 3. Materials and Methods

### 3.1. Materials

**Figure 5:**
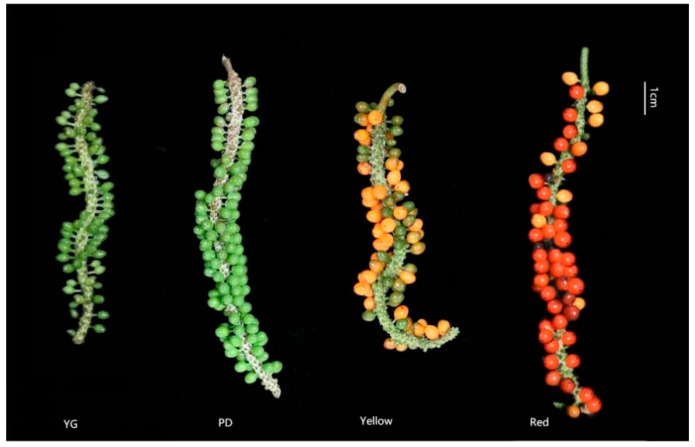
Fruits at the young (YG), expanding (PD), yellow (Yellow), and red fruit stages (Red).

Fresh young (60 days after pollination), expanding (90 days after pollination), yellow (120 days after pollination), and red fruits (150 days after pollination) of *P. laetispicum* were collected from a germplasm nursery at the Institute of Spices and Beverage Research, the Chinese Academy of Tropical Agricultural Sciences, Hainan, China. The four developmental stages were recorded as YG, PD, Yellow, and Red (Figure 5). After being collected, the fruits were washed, dried, ground, screened, and then stored in closed hermetic bags at 25 °C.

### 3.2. Crude Fat Determination

The crude fat content was determined by using ultrasonic-assisted extraction. Briefly, 3 g (M_0_) of *P. laetispicum* fruit powder was put into a centrifuge tube (M_2_), 27 mL of petroleum ether was added, and the mixture was then placed into a 25 °C ultrasonic machine for 20 min. It was then centrifuged at 8000 r/min and 25 °C for 10 min. Ultrasound treatment and centrifugation were performed twice. The supernatant was taken, the excess petroleum ether was volatised, and the precipitate was dried in a 95 °C drum for 4 h. It was then weighed after cooling (M_1_). The crude fat content was calculated using Equation (1) (mass accurate to the tens of thousands):

(1) $$X=\frac{\left(M_{1}-M_{2}\right)}{M_{0}}\times 100\%$$

where X represents the percentage of crude fat, M_1_ denotes the centrifuge tube and residual masses, M_2_ indicates the centrifugal tube weight, and M_0_ represents the *P. laetispicum* powder weight.

### 3.3. Total Ash Content Measurement

The total ash content was determined based on the National Standard [35]. Briefly, 2 g of *P. laetispicum* powder was put into a crucible and then placed on an electric stove to carbonise the sample. After, it was baked in a furnace at 550 °C for 4 h. The ash left in the crucible was weighed.

### 3.4. Determination of Piperine Level

The piperine content was measured based on the National Standard [36]. Briefly, 3 g of *P. laetispicum* powder was heated at 80 °C for 3 h before being put into a 100 mL volumetric bottle. Then, 3 mL was placed into a 25 mL volumetric bottle, and the content of piperine was detected using high-performance liquid chromatography (HPLC). The HPLC conditions were as follows: We used an Eclipse XDB C18 column (4.6 mm × 250 mm, 5 μm) (Agilent Technologies, Palo Alto, CA, USA); a mixture of methanol and water at a volume ratio of 77:23 was used as a mobile phase; the column temperature was 30 °C; the flow rate was 1.0 mL/min; the detection wavelength was 343 nm; and the sample size was 10 μL. The piperine standard concentration gradients were 0.4 mg/mL, 0.8 mg/mL, 1.2 mg/mL, 1.6 mg/mL, and 2.0 mg/mL. A standard curve was drawn, and the results were calculated.

### 3.5. Steam Distillation Combined with GC-MS Analysis of Volatile Oil Compound (VOCs)

The fruit essential oil was extracted using steam distillation [37]. Briefly, 30 g of *P. laetispicum* powder was added to 300 mL of pure water. Distillation was then performed under continuous boiling conditions (approximately five drops per minute, dripped from the condenser) for 3 h, and the essential oil was collected. Hexane was diluted 200 times and detected using GC-MS.

The GC conditions were as follows: We used TG-WAXMS (30 m × 0.25 mm × 0.25 m) (Thermerscheer Technology, Shanghai, China); splitless injection was performed; the injection port temperature was 250 °C; the injection volume was 0.5 uL; the solvent delay was 4 min; the carrier (helium) purity was 99.999%; and the flow rate was 1 mL/min. The initial column temperature was 40 °C, which was maintained for 5 min. It was then increased to 220 °C at a rate of 4 °C/min, maintained for 5 min, and then increased to 300 °C at 10 °C/min for 5 min.

The MS conditions were as follows: the electron impact energy was 70 eV; the ion source temperature was 230 °C; the transmission line temperature was 300 °C; and the scanning range was 30-400 amu.

An NIST14 spectral library search and retention index qualitative determination were conducted to identify the volatile compounds. The area normalisation approach was used to calculate the relative content of each of the chemicals. The formula for calculating the linear retention index (LRI), where the number of carbon atoms in n-alkanes was C7–C30, was as follows:

$$LRI=100\times (n+\frac{t-t_{n}}{t_{n+1}-t_{n}})$$

where n and n + 1 represent the number of carbon atoms of n-alkane before and after the compound was measured, respectively. t_n_ and t_n+1_ denote the peak retention time of the corresponding n-paraffin, and this was the peak retention time of the component that was measured (t_n_ < t < t_n+1_).

### 3.6. Solid-Phase Extraction Combined with GC-MS for the Analysis of Aroma Metabolites

Metabolite extraction: The fruits were placed into a sterile centrifuge tube, and then quickly frozen in liquid nitrogen for 15 min. They were then stored at −80 °C for later use. Firstly, an appropriate amount of sample was added to a 20 mL headspace vial, and 4 mL saturated sodium chloride aqueous solution was added. Then, 10 μL of an internal standard solution was added to each sample and then incubated at 60 °C for 10 min. An SPME fibre was put into a chamber at 270 °C for 10 min before each sample was extracted. The SPME was incubated at 60 °C for 40 min, and it was desorbed at 250 °C for 5 min in a GC injector. Finally, the SPME fibre was put in a chamber at 270 °C for 10 min after the injection step.

GC-MS analysis: Analyses were conducted using a LECO Pegasus^®^ 4D instrument (LECO, St. Joseph, MI, USA) consisting of an Agilent 8890A GC (Agilent Technologies, Palo Alto, CA, USA) system equipped with a split/spitless injector and a dual-stage cryogenic modulator (LECO) coupled with a TOF-MS detector (LECO). An Rxi-5Sil MS (30 m × 250 μm I.D., 0.5 μm) (Restek, Bellefonte, PA, USA) was used as the first-dimension column (1 D) and an Rxi-17Sil MS (2.0 m × 150 μm I.D., 0.15 μm) (Restek, USA) was used as the second-dimension column (2 D). The carrier gas was high-purity helium (>99.999%) with a constant flow rate of 1.0 mL/min. The oven temperature was maintained at 50 °C for 1 min and then raised to 170 °C at a rate of 2 °C/min and held for 1 min. It was then raised to 230 °C at a rate of 30 °C/min and maintained for 1 min. The second oven temperature was 5 °C higher than that of the first oven. The temperature of the modulator was 3 °C higher than the temperature of the second column. The modulator was operated with a 0-s modulation period and the GC injector temperature was 250 °C.

The acquisition frequency was 20 spectra/s. The mass spectrometer was operated in the EI mode at 70 eV with a range of *m/z* 35–550 and a detector voltage of 1984 V.

### 3.7. Statistical Analysis

All the experiments were repeated three times, and the results are presented as means ± standard deviation (SD). The data were analysed using one-way ANOVA, followed by Spass to determine the differences between the samples (*p* < 0.05). Following Excel 2021 selection and the treatment of detected volatiles, principal component analysis (PCA) was performed, and stacked bar charts were created using Origin2022. TBtools was used for hierarchical clustering. The analysis of aroma metabolite raw data, including peak extraction, baseline adjustment, deconvolution, alignment and integration, was performed using Chroma TOF (V4.72.0.0) software, and the NIST2017 database was used for metabolite identification by matching the mass spectrum and retention index.

## 4. Conclusions

*P. laetispicum* was found to have high contents of protein and fat, and it was rich in VOCs and metabolites. VOCs can be divided into alcohols, alkenes, ketones, esters, and benzenes based on their functional groups. The principal component analysis found that alcohols, alkenes, and esters contributed the most to PC1. Non-targeted metabolome analysis found the metabolites to be the most abundant in linalool. There were more different aromatic metabolites from the young to expanding fruit stages. The contents of protein, crude fat, and ash in the young fruit stage were higher than those in the other three growth stages. The differences in the VOCs and aroma metabolites between the young and red fruit stages were the greatest. Nutrient composition is the carrier of many drugs’ metabolism in the human body, and too few nutrients affect the human body’s ability to accept drugs. In this study, the nutrient content of young fruit was higher, so it can be better utilised. Most of the aroma components of *P. laetispicum* have medicinal effects and can be used to extract essential oils. The four fruit stages were dominated by alkenes. The relative content of alcohols in the young fruit stage and the relative content of alkenes in the red fruit stage were the highest in the four fruit stages. The extraction of oil from fruits at different growth stages is essential. The results of this study demonstrate the differences in the nutrients and aroma components in the four developmental stages of *P. laetispicum* fruit. This study may accelerate subsequent research on the effective utilisation of *P. laetispicum*.

## Figures and Tables

**Table 1:** The contents of crude fat, ash, piperine and protein at different stages.

Matter (%)	YG	PD	Yellow	Red
Crude fat	13.9	14.34	11.15	7.31
Ash	10.97	9.56	6.59	6.89
Piperine	0.00	0.00	0.08	0.00
Protein	20.24	19.57	11.57	13.71

**Table 2:** Components and contents of volatile oil compounds (VOCs) at four developmental stages.

No.	Category	LRI	Type ID	Component	Relative Percentage (%) ± SD Percentage			
					YG	PD	Yellow	Red
1	Alcohols	1084.48	Alcohols1	Trans-*β*-Terpineol	0.19 ± 0.07 ^a^	0.49 ± 0.32 ^a^	0.14 ± 0.00 ^a^	0.09 ± 0.03 ^a^
2		1624.20	Alcohols2	Linalool	15.70 ± 2.43 ^a^	8.54 ± 0.00 ^b^	6.24 ± 0.16 ^b^	2.42 ± 0.02 ^c^
3		1624.20	Alcohols3	(E)-p-2-menthen-1-ol	0.10 ± 0.03 ^a^	0.06 ± 0.00 ^ab^	——	0.03 ± 0.00 ^b^
4		1140.63	Alcohols4	4-terpineol	2.87 ± 0.37 ^a^	1.94 ± 0.06 ^b^	0.70 ± 0.01 ^c^	0.75 ± 0.01 ^c^
5		1624.20	Alcohols5	*α*-terpineol	0.30 ± 0.07 ^a^	0.18 ± 0.10 ^ab^	0.06 ± 0.00 ^c^	0.04 ± 0.00 ^c^
6		1296.34	Alcohols6	(*Z*)-linalool oxide (furanoid)	0.13 ± 0.05 ^a^	——	——	——
7		1624.20	Alcohols7	*α*-bisabolol	0.21 ± 0.17 ^a^	——	——	——
8	Alkenes	936.87	Alkenes1	(−)-*α*-pinene	0.57 ± 0.07 ^a^	0.33 ± 0.02 ^b^	0.33 ± 0.01 ^b^	——
9		979.49	Alkenes2	Sabinene	34.83 ± 8.19 ^b^	67.52 ± 0.43 ^a^	70.64 ± 1.24 ^a^	76.14 ± 0.38 ^a^
10		997.47	Alkenes3	*β*-pinene	0.62 ± 0.28 ^a^	——	——	0.24 ± 0.01 ^a^
11		1010.10	Alkenes4	*α*-phellandrene	0.15 ± 0.07 ^a^	0.08 ± 0.00 ^a^	0.11 ± 0.01 ^a^	0.06 ± 0.00 ^a^
12		1022.66	Alkenes5	Terpinolene	0.29 ± 0.15 ^a^	0.09 ± 0.02 ^ab^	0.06 ± 0.01 ^b^	0.10 ± 0.00 ^ab^
13		1036.45	Alkenes6	*α*-pinene	3.72 ± 1.73 ^a^	2.16 ± 0.12 ^a^	1.51 ± 0.85 ^a^	4.31 ± 0.08 ^a^
14		1067.00	Alkenes7	γ-terpinene	0.96 ± 0.22 ^a^	0.25 ± 0.00 ^b^	0.06 ± 0.00 ^b^	0.19 ± 0.02 ^b^
15		1347.75	Alkenes8	1-ethenyl-1-methyl-2,4-bis(1-methylethylidene)-cyclohexane	0.61 ± 0.06 ^a^	0.33 ± 0.01 ^b^	0.56 ± 0.10 ^a^	0.12 ± 0.00 ^c^
16		1390.39	Alkenes9	*α*-copaene	19.51 ± 3.52 ^a^	10.53 ± 0.17 ^b^	7.06 ± 0.03 ^bc^	5.11 ± 0.02 ^c^
17		1402.87	Alkenes10	*β*-cubebene	1.36 ± 0.89 ^a^	0.32 ± 0.01 ^a^	0.95 ± 0.02 ^a^	0.09 ± 0.10 ^a^
18		1407.01	Alkenes11	1-ethenyl-1-methyl-2,4-bis(1-methylethenyl)-cyclohexane	0.32 ± 0.24 ^a^	0.20 ± 0.16 ^a^	0.12 ± 0.01 ^a^	0.04 ± 0.00 ^a^
19		1436.62	Alkenes12	trans-caryophyllene	6.57 ± 0.21 ^a^	3.38 ± 0.00 ^c^	4.20 ± 0.12 ^b^	2.37 ± 0.01 ^d^
20		1448.41	Alkenes13	(E)-*α*-bergamotene	0.99 ± 0.56 ^a^	——	0.16 ± 0.00 ^a^	0.06 ± 0.00 ^a^
21		1452.23	Alkenes14	Aromadendrene	0.06 ± 0.03 ^a^	0.12 ± 0.07 ^a^	0.05 ± 0.03 ^a^	——
22		1472.29	Alkenes15	*α*-caryophyllene	0.88 ± 0.04 ^b^	0.44 ± 0.01 ^c^	1.19 ± 0.06 ^a^	0.51 ± 0.02 ^c^
23		1494.59	Alkenes16	*α*-amorphene	0.11 ± 0.09 ^a^	0.12 ± 0.08 ^a^	0.06 ± 0.02 ^a^	0.02 ± 0.00 ^a^
24		1500.00	Alkenes17	Germacrene D	3.74 ± 1.32 ^a^	1.30 ± 0.29 ^b^	4.10 ± 0.32 ^a^	3.64 ± 0.05 ^a^
25		1518.92	Alkenes18	*α*-muurolene	0.31 ± 0.16 ^a^	——	——	0.06 ± 0.00 ^a^
26		1525.34	Alkenes19	*β*-bisabolene	0.35 ± 0.01 ^a^	0.16 ± 0.01 ^b^	0.12 ± 0.01 ^c^	0.10 ± 0.00 ^c^
27		1542.91	Alkenes20	(+)-δ-cadinene	3.69 ± 0.22 ^a^	1.49 ± 0.07 ^bc^	1.22 ± 0.06 ^c^	1.65 ± 0.02 ^b^
28		998.16	Alkenes21	*β*-myrcene	——	0.13 ± 0.00 ^a^	0.13 ± 0.00 ^a^	——
29		1347.45	Alkenes22	Santolina triene	——	0.16 ± 0.11 ^a^	——	——
30		925.35	Alkenes23	4-methyl-1-(1-methylethyl) bicyclo[3.1.0]hexane didehydro deriv	——	——	——	1.42 ± 0.01 ^a^
31		1686.12	Alkenes24	Guaiazulene	——	——	——	0.01 ± 0.00 ^a^
32	Esters	1239.44	Esters1	2-ethylhexyl acrylate	0.10 ± 0.02 ^a^	0.06 ± 0.00 ^a^	0.08 ± 0.00 ^a^	——
33	Ketones	1293.80	Ketones1	2-undecanone	0.52 ± 0.23 ^a^	0.14 ± 0.00 ^b^	0.13 ± 0.01 ^b^	0.05 ± 0.00 ^b^
34	Benzenes	1025.12	Benzenes1	o-cymene	——	——	——	0.10 ± 0.01 ^a^

Different lowercase letters in the same column indicate significant differences between treatments (*p* < 0.05). ——, not detected.

**Table 3:** The eigenvalues and variance contributions of the four principal components.

Principal Component	Initial Eigenvalues			Extraction Sums of Squared Loadings		
	Total	Variance Contribution Rate (%)	Cumulative (%)	Total	Variance Contribution Rate (%)	Cumulative (%)
1	26.79	54.67	54.67	26.79	54.67	54.67
2	13.90	28.36	83.02	13.90	28.36	83.02
3	8.32	16.98	100.00	8.32	16.98	100.00

**Table 4:** Principal component loading matrix.

VOCs	PC1	PC2	PC3
Alcohols2	0.998	−0.019	−0.064
Alcohols4	0.914	0.098	−0.393
Alcohols5	0.949	0.038	−0.314
Alcohols9	0.937	0.134	−0.322
Alcohols6	0.908	0.410	0.089
Alcohols7	0.908	0.410	0.089
Alcohols11	−0.025	−0.581	−0.814
Alcohols12	−0.197	−0.553	0.81
Alcohols14	−0.686	0.723	−0.085
Alkenes1	0.949	−0.293	0.112
Alkenes2	−0.971	−0.239	−0.03
Alkenes4	0.938	−0.027	0.345
Alkenes5	0.859	0.511	−0.042
Alkenes6	0.026	0.976	−0.216
Alkenes7	0.895	0.433	−0.109
Alkenes9	0.984	0.132	−0.122
Alkenes11	0.980	−0.081	−0.179
Alkenes25	0.909	0.403	0.105
Alkenes12	0.956	0.036	0.290
Alkenes15	0.291	−0.273	0.917
Alkenes16	0.772	−0.479	−0.419
Alkenes17	0.022	0.447	0.894
Alkenes19	0.964	0.263	−0.036
Alkenes20	0.857	0.515	−0.014
Alkenes21	−0.195	−0.98	0.029
Esters1	0.854	−0.43	0.293
Esters2	−0.197	−0.553	0.810
Ketones1	0.969	0.232	0.085
Ketones3	−0.686	0.723	−0.085
Benzenes1	−0.686	0.723	−0.085

## Data Availability

The data are contained within this article.

## Appendix A

**Table A1:** Content table of volatile oil compounds in *P. laetispicum* fruit at four developmental stages (few aroma compounds).

No.	Category	LRI	Type ID	Component	Relative Percentage (%) ± SD Percentage			
					YG	PD	Yellow	Red
1		1160.95	Alcohols8	(*Z*)-p-2-menthen-1-ol	0.08 ± 0.02 ^a^	0.04 ± 0.00 ^a^	——	0.02 ± 0.00 ^a^
2		1230.99	Alcohols9	(*Z*)-piperitol	0.03 ± 0.01 ^a^	0.02 ± 0.00 ^a^	——	——
3		1602.14	Alcohols10	(+)-viridiflorol	0.04 ± 0.00 ^a^	——	——	0.02 ± 0.00 ^a^
4		1623.13	Alcohols11	Nerolidol	——	0.03 ± 0.01 ^a^	——	——
5		1585.47	Alcohols12	(±)-trans-nerolidol	——	——	0.05 ± 0.00 ^a^	——
6		1584.46	Alcohols13	1,7-dimethyl-4-propan-2-ylcyclodeca-2,7-dien-1-ol	——	——	——	0.01 ± 0.00 ^a^
7		1656.94	Alcohols14	*α*-muurolol	——	——	——	0.01 ± 0.00 ^a^
8		1421.97	Alkenes25	(−)-*α*-gurjunene	0.06 ± 0.04 ^a^	0.01 ± 0.00 ^b^	0.01 ± 0.00 ^b^	0.01 ± 0.00 ^b^
9		1609.61	Alkenes26	Caryophyllene oxide	0.07 ± 0.01 ^a^	——	0.06 ± 0.01 ^a^	0.07 ± 0.00 ^a^
10		945.62	Alkenes27	Camphene	——	——	——	0.03 ± 0.00 ^a^
11		1348.95	Alkenes28	(−)-*α*-cubebene	——	——	——	0.08 ± 0.00 ^a^
12		1438.85	Alkenes29	*α*-guaiene	——	——	——	0.01 ± 0.00 ^a^
13		1264.51	Esters2	Linalyl anthranilate	——	——	0.02 ± 0.00 ^a^	——
14		1184.43	Ketones2	2,6,6-trimethyl-2,4-Cycloheptadien-1-one	——	——	0.02 ± 0.00 ^a^	——
15		1172.96	Ketones3	Umbellulon	——	——	——	0.01 ± 0.00 ^a^

Different lowercase letters in the same column indicate significant differences between treatments (*p* < 0.05). ——, not detected.

**Table A2:** Table of fruit aroma content components of *P. laetispicum* at developmental stage detected by using non-targeted metabolome (Some major metabolites).

No.	Type ID	RT (min)	YG	PD	Yellow	Red
1	α-pinene, (+)-	5.2686	2.61 ± 0.15 ^c^	3.81 ± 0.23 ^b^	3.91 ± 0.27 ^b^	5.04 ± 0.31 ^a^
2	(−)-β-pinene	7.38094	0.74 ± 0.05 ^b^	2.85 ± 0.19 ^a^	3.26 ± 0.38 ^a^	5.09 ± 0.49 ^a^
3	Sabinene	8.0155	1.61 ± 0.60 ^a^	10.26 ± 0.88 ^b^	9.18 ± 1.85 ^b^	8.45 ± 3.33 ^ab^
4	Trans-2-pentenal	8.30661	0.02 ± 0.00 ^a^	0.01 ± 0.00 ^a^	0.01 ± 0.00 ^a^	0.00 ± 0.00 ^a^
5	Myrcene	9.14548	1.81 ± 0.08 ^ab^	1.07 ± 0.32 ^bc^	1.07 ± 0.32 ^c^	1.14 ± 0.38 ^a^
6	α-cubebene	18.4893	2.04 ± 0.13 ^a^	0.71 ± 0.32 ^b^	0.87 ± 0.52 ^ab^	1.57 ± 0.96 ^ab^
7	Copaene	19.3107	9.11 ± 0.79 ^a^	6.07 ± 1.63 ^a^	4.02 ± 2.73 ^a^	10.65 ± 1.29 ^a^
8	Linalool	20.7709	17.17 ± 0.54 ^ab^	12.18 ± 3.20 ^b^	14.05 ± 0.98 ^a^	15.36 ± 1.04 ^ab^
9	α-bergamotene, (e)-(−)-	21.7403	1.17 ± 0.13 ^b^	1.05 ± 0.36 ^b^	1.52 ± 0.73 ^b^	2.17 ± 2.27 ^a^
10	4-terpineol, (−)-	21.7968	0.86 ± 0.25 ^a^	2.28 ± 1.20 ^ab^	1.82 ± 0.59 ^b^	0.51 ± 0.00 ^b^
11	2-undecanone	21.87925	4.43 ± 0.14 ^a^	3.90 ± 0.07 ^a^	4.59 ± 0.34 ^a^	4.04 ± 0.28 ^a^
12	β-caryophyllene	21.9228	1.66 ± 0.20 ^c^	2.24 ± 0.29 ^bc^	1.62 ± 0.8 b	4.02 ± 0.57 ^a^
13	α-terpineol	23.8872	1.14 ± 0.09 ^a^	0.95 ± 0.69 ^a^	0.24 ± 0.15 ^a^	0.17 ± 0.15 ^a^
14	Gamma-muurolene	23.97825	1.14 ± 0.09 ^a^	0.47 ± 0.22 ^a^	0.97 ± 0.64 ^a^	0.62 ± 0.24 ^a^
15	Germacrene D	24.3739	1.31 ± 0.21 ^ab^	3.71 ± 0.84 ^a^	6.00 ± 2.54 ^ab^	12.68 ± 2.02 ^b^
16	Bicyclogermacren	24.9042	1.97 ± 0.37 ^a^	2.92 ± 0.05 ^b^	1.94 ± 1.23 ^b^	2.66 ± 1.19 ^a^
17	β-cadinene	25.6386	6.53 ± 0.54 ^a^	4.77 ± 2.00 ^ab^	7.64 ± 3.11 ^b^	8.3 ± 3.50 ^b^

Different lowercase letters in the same column indicate significant differences between treatments (*p* < 0.05).
